# Luminal-contact-inhibition of epithelial basal stem cell multipotency in prostate organogenesis and homeostasis

**DOI:** 10.1242/bio.045724

**Published:** 2019-09-20

**Authors:** Corrigan Horton, Yueli Liu, Chuan Yu, Qing Xie, Zhu A. Wang

**Affiliations:** Department of Molecular, Cell, and Developmental Biology, University of California, Santa Cruz, CA 95064, USA

**Keywords:** Prostate stem cell, Plasticity, Basal, Luminal, Lineage tracing

## Abstract

Prostate epithelial basal cells are highly plastic in their luminal differentiation capability. Basal stem cells actively produce luminal cells during organogenesis, but become restricted in the adult prostate unless receiving oncogenic or inflammatory stimuli. Given that the number of luminal cells increases relative to basal cells through development and that equilibrium is reached in the adulthood, we hypothesize that a negative-feedback mechanism exists to inhibit basal-to-luminal differentiation. We provide evidence supporting this hypothesis by comparing murine prostatic growth in a tissue reconstitution assay with cell recombinants of different basal-to-luminal ratios. Additionally, in organoid culture, hybrid organoids derived from adjacent basal and luminal cells showed reduced basal stem cell activities, suggesting contact inhibition. Importantly, removal of adult luminal cells *in vivo* via either an inducible Cre/*loxP*-Dre/*rox* dual-lineage-tracing system or orthotopic trypsin injection led to robust reactivation of basal stem cell activities, which acts independent of androgen. These data illustrate the prostate organ as a distinctive paradigm where cell contact from differentiated daughter cells restricts adult stem cell multipotency to maintain the steady-state epithelial architecture.

## Abstract (precis)

**Summary:** Lineage tracing shows that ablation of prostate luminal cells reactivates adult basal cell multipotency, while luminal–basal cell contact inhibits basal stem cell activities in organoids.

## INTRODUCTION

The cell lineage relationship and cell fate transitions in the prostate gland have been under intense investigation recently, because the knowledge is crucial for understanding both prostate development and cancer progression (Davies et al., 2018; Le Magnen et al., 2018; Toivanen and Shen, 2017). The stratified prostate epithelium is comprised of a basal cell layer, a layer of secretory luminal cells, and rare interspersed neuroendocrine cells. In both human and mouse prostate, basal and luminal cells are distinguishable by their different morphologies and molecular marker profiles. Basal cells are triangular or flat-shaped, residing near the basement membrane and expressing marker cytokeratin (CK) 5. In contrast, luminal cells are column-like, residing at the apical side of the epithelium and expressing marker CK18, as well as high levels of the transcription factor Nkx3.1 (Toivanen and Shen, 2017; Xie and Wang, 2017).

Strong evidence supports basal cells behaving as stem cells to generate luminal cells in prostate development. First, epithelial cells in the budding prostate initially show a CK5^+^CK18^+^ intermediate cell phenotype before a luminal-specific layer is specified (Toivanen and Shen, 2017; Wang et al., 2001). In this process, basal cells display both symmetrical and asymmetrical divisions leading to different cell fates, while luminal cells only exhibit symmetrical divisions (Wang et al., 2014). Second, basal cells consistently perform better than luminal cells in assays mimicking prostate organogenesis, including the prostate sphere and organoid culture (Chua et al., 2014; Goldstein et al., 2008; Karthaus et al., 2014; Lawson et al., 2007), and the renal graft-based tissue reconstitution assay (Goldstein et al., 2010; Wang and Shen, 2011; Xin et al., 2003). Finally, mouse genetic lineage-tracing analyses using basal-specific Cre drivers demonstrate that neonatal basal cells efficiently generate luminal cells during postnatal development *in vivo* (Ousset et al., 2012; Pignon et al., 2013; Wuidart et al., 2016). Recently, sporadic mitochondrial DNA mutations were used to trace human prostate tissues and the data also supported the existence of multipotent basal stem cells (Moad et al., 2017).

Interestingly, basal stem cell functions are highly plastic. Tracing of adult basal cells showed that they are mostly lineage restricted, as basal-to-luminal differentiation is very rare in the mature organ (Choi et al., 2012; Wang et al., 2013). Basal cell plasticity is further demonstrated by their enhanced luminal differentiation under oncogenic (Choi et al., 2012; Lu et al., 2013; Wang et al., 2013) and inflammatory conditions (Kwon et al., 2013). We recently showed that the cell-autonomous androgen receptor is required for basal-to-luminal cell differentiation (Xie et al., 2017), but the mechanism of basal cell plasticity remains poorly understood. Several cues led us to hypothesize that differentiated luminal cells negatively regulate basal stem cell multipotency. First, as more luminal cells are produced, the frequency of basal-to-luminal differentiation decreases through development. Second, purified basal cells appeared to have higher sphere-forming efficiency compared to their counterparts within an unsorted total cell population (Wang et al., 2013). Third, luminal cell anoikis resulting from E-Cadherin loss can lead to an increase of basal cell proliferation, although basal-to-luminal differentiation has not been definitively shown by lineage tracing (Toivanen et al., 2016). Here, we tested the hypothesis in prostate development using organoid and tissue reconstitution assays, and in the adult prostate by lineage tracing. Our results support a model in which direct basal–luminal cell contact is an essential negative regulator of prostate basal cell bipotentiality.

## RESULTS

### Luminal cells inhibit prostatic growth from basal cells in tissue reconstitution assay

**Fig. 1.**
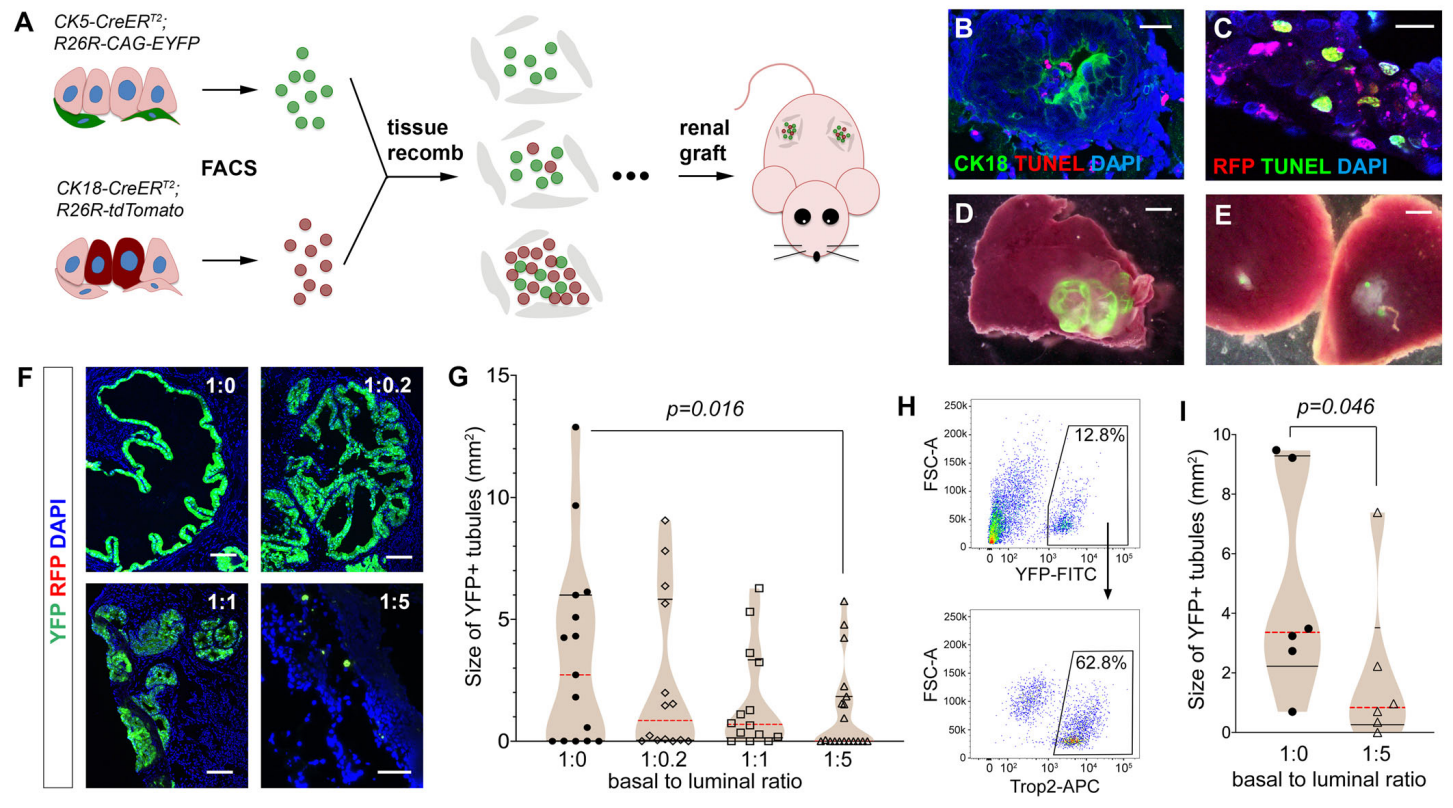
**Analyzing the effects of mixing basal and luminal cells in prostate tissue reconstitution assay.** (A) Diagram of renal grafting experiments with different ratios of basal and luminal cells. (B,C) IF and TUNEL staining of day 7 renal graft showing apoptotic signals in a fraction of luminal cells marked by CK18^+^ (B) or RFP^+^ (C). Scale bars: 20 μm. (D,E) YFP and white field overlay dissection images showing a graft of the 1:0.2 ratio (D) and grafts of the 1:5 ratio (E). Scale bars: 1 mm. (F) Representative images showing direct visualization for YFP and RFP signals in sectioned grafts. Scale bars: 100 μm. (G) Violin plot comparing YFP^+^ tubule sizes for all the grafts with different basal-to-luminal ratios by Mann–Whitney *U*-test. Black lines show quartiles and red dashed lines show medians. (H) FACS gating for sorting Trop2^+^ basal cells. (I) Violin plot comparing sizes of YFP^+^ tubules derived from Trop2^+^ basal cells in the cell mixture experiment by Mann–Whitney *U*-test.

To test whether there is causality between the increasing number of luminal cells and decrease of basal cell plasticity during prostate development, we mixed fluorescence-labeled luminal and basal cells at different ratios, and analyzed the growth of prostatic tissues using the tissue reconstitution assay (Fig. 1A). As we have done previously (Xie et al., 2017), total basal cells were obtained by flow-sorting of YFP^+^ cells from *CK5-CreER^T2^; R26R-CAG-YFP/+* mice that were tamoxifen-induced at 8 weeks of age (Fig. S1A). To isolate luminal cells, we flow-sorted RFP^+^ cells from tamoxifen-induced *CK18-CreER^T2^; Ai9/+* mice (Madisen et al., 2010; Van Keymeulen et al., 2009) (Fig. S1B), in which luminal cells were specifically marked by tdTomato upon induction (Fig. S1C). We then mixed the two sorted cell populations at basal-to-luminal ratios of 1:0, 1:0.2, 1:1, and 1:5, to mimic the epithelial cell composition at various developmental stages from prostate budding to adulthood. The mixed cells were recombined with rat urogenital sinus mesenchyme (UGSM) cells and grafted under the renal capsule of nude mice. Since the renal grafting assay is not conducive to prostatic tissue growth from luminal cells (Lukacs et al., 2010; Xin et al., 2003), we fixed the basal cell number at 5000 in each cell recombinant, so that the influence of luminal cell number on basal cell activities could be compared. Mixed basal and luminal cells organized into small tubules within 7 days of growth (Fig. 1B,C). TUNEL staining revealed that most basal cells were not apoptotic in the grafts, while ∼40% of luminal cells already showed positive signals by 1 day of growth, and luminal apoptosis persisted at 7 days (Fig. 1B,C; Fig. S1D). These data suggest that grafted luminal cells were continuously being eliminated due to unfavorable assay conditions. After 2 months, most of the grown grafts were YFP^+^, and RFP^+^ cells were not found (Fig. 1D,F), confirming their basal cell origin. On the other hand, small dots of YFP+ signals could be found in grafts that failed to grow (Fig. 1E,F). We then tested whether the grafted luminal cells, while alive, had any effect on basal-derived prostatic tissues by measuring the sizes of the YFP^+^ tubules 2 months after grafting. We found a trend of higher luminal fraction correlating with smaller prostatic size (Fig. 1F,G). Notably, the 1:5 group contained significantly smaller grafts than the 1:0 group (*P*=0.016 by the Mann–Whitney *U*-test), as many more grafts failed to grow (Fig. 1G). The median sizes in the 1:0.2 and 1:1 groups were also smaller than the pure basal group, although the differences did not reach significance. These data support that luminal cells negatively affect basal cell plasticity during prostate organogenesis. Since YFP^+^ basal cells represent the total basal population (Xie et al., 2017), we next tested whether the effect can be observed by using the Trop2^+^ basal cells (Fig. 1H), a stem cell population enriched for robust renal graft activity (Goldstein et al., 2008). Indeed, adding luminal cells significantly suppressed tubule growth from Trop2^+^ basal cells (Fig. 1I), confirming they directly inhibit basal stem cells in organogenesis.

### Luminal-basal cell contact suppresses basal cell bipotentiality in organoids

**Fig. 2.**
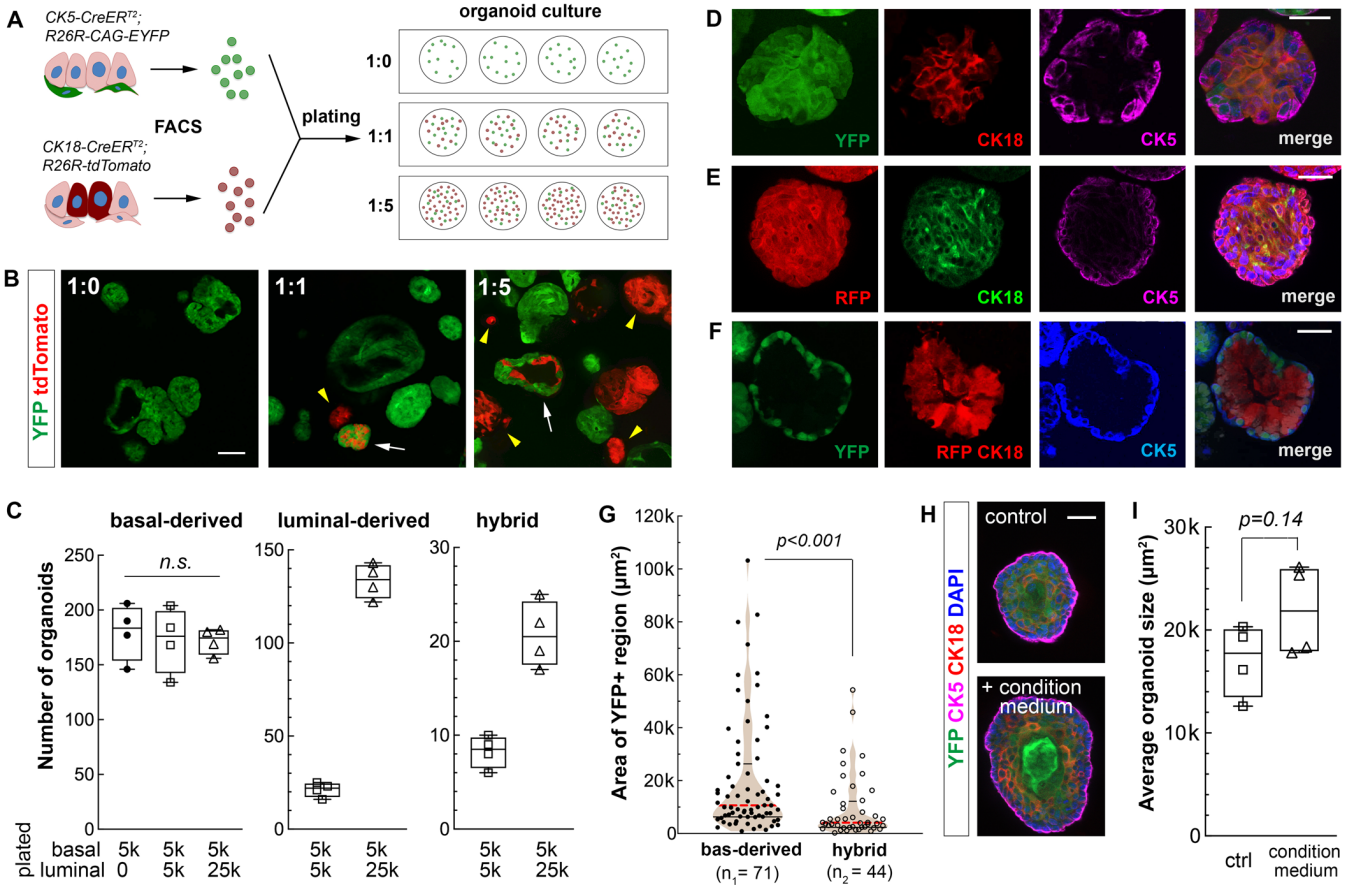
**Effects of co-culturing luminal cells on basal stem cell activities in prostate organoid assay.** (A) Diagram of organoid culture experiments plated with different ratios of basal and luminal cells. (B) Direct visualization of YFP and RFP signals in organoids under different ratio conditions. White arrows, hybrid organoids. Yellow arrowheads, luminal-derived organoids. Scale bar: 100 μm. (C) Box plot comparing organoid numbers for each type per well (*N*=4) under different basal-luminal mixture conditions. n.s., not significant by Student's *t*-test. (D–F) IF images showing basal-to-luminal differentiation in a basal-derived organoid (D), luminal-to-basal differentiation in a luminal-derived organoid (E), and cell lineage restriction in a hybrid organoid (F). Scale bars: 50 μm. (G) Violin plot comparing YFP^+^ areas between basal-derived organoids and hybrid organoids by Mann–Whitney *U*-test. Black lines show quartiles and red dashed lines show medians. (H) Representative IF images showing basal-derived organoid cultured with standard medium or luminal-conditioned medium. Scale bar: 50 μm. (I) Box plot comparing average size of basal-derived organoid per well (*N*=4) with and without luminal-conditioned medium by Student's *t*-test.

The luminal inhibitory effect could be due to luminal-secreted paracrine signals. In the case of renal graft assay, apoptotic luminal cells might send death signals. Alternatively, luminal cells may inhibit basal cells through direct cell contact. To distinguish between these two possibilities, we resorted to prostate organoid culture (Drost et al., 2016; Karthaus et al., 2014) for cell mixing experiments. This assay models prostate organogenesis *in vitro*, but it offers high resolution of prostatic tubule growth from individual cells and, unlike the renal graft assay, is conducive to luminal cell growth (Chua et al., 2014; Karthaus et al., 2014). YFP^+^ basal cells and RFP^+^ luminal cells were sorted and plated in 1:0 (pure basal), 1:1, and 1:5 ratios under organoid culture conditions. Basal cell number was fixed at 5000 in each of the four wells of each ratio (Fig. 2A). Eight days after culture, YFP^+^ basal-derived organoids were present in all the wells, while the 1:1 and 1:5 wells also contained RFP^+^ luminal-derived organoids as well as hybrid organoids that comprised intermingled YFP^+^ and RFP^+^ cells (Fig. 2B). The numbers of each type of organoids were quantified for each ratio, and adding luminal cells did not affect the formation efficiency of basal-derived organoids (Fig. 2C). As expected, CK5 and CK18 staining revealed that extensive basal-to-luminal cell differentiation occurred in pure basal-derived organoids (Fig. 2D), and vice versa in pure luminal-derived organoids (Fig. 2E). No neuroendocrine cells were detected. In contrast, the hybrid organoids likely developed from coordinated actions of single basal and luminal cells plated in close proximity, as they usually contained YFP^+^ cells on the outer layer surrounding inner RFP^+^ cells (Fig. 2F). Interestingly, those YFP+ cells remained CK5+ and the RFP+ cells remained CK18+ (Fig. 2F), indicating cell lineage conversion is inhibited when the two cell types are in close contact. Furthermore, when we compared the sizes of YFP^+^ regions between basal-derived organoids and hybrid organoids, we found that those of the hybrid organoids were significantly smaller (*P*<0.001 by the Mann–Whitney *U*-test) (Fig. 2G), indicating that basal stem cells on average underwent fewer rounds of cell division in the hybrids.

To directly test whether luminal cells secrete any inhibitory factors, we cultured pure RFP^+^ luminal cells in the organoid assay for 4 days, and transferred the condition medium to pure basal organoid culture. Compared to controls using standard medium, adding the luminal condition medium actually slightly increased the size and luminal differentiation (Fig. 2H,I), suggesting luminal inhibitory effects are not mediated in a paracrine fashion. Finally, to test whether basal–basal cell contact has any inhibitory effect, we obtained unmarked average basal cells by sorting from wild-type mice using cell surface markers Lin^−^Sca-1^+^CD49f^hi^ (Lawson et al., 2007; Lukacs et al., 2010) (Fig. S2A). We then mixed YFP^+^ basal cells with these unmarked basal cells in 1:5 ratio in the organoid assay (Fig. S2B). In the resulting green-white basal hybrid organoids, we frequently observed basal-to-luminal differentiation by CK5 and CK18 staining (Fig. S2C). Moreover, the sizes of YFP^+^ regions in those hybrids were not significantly different from pure YFP^+^ organoids (Fig. S2D). Taken together, our organoid results support that basal stem cell activities are inhibited by direct luminal cell contact.

### Increased basal cell proliferation upon luminal cell ablation in the adult prostate

**Fig. 3.**
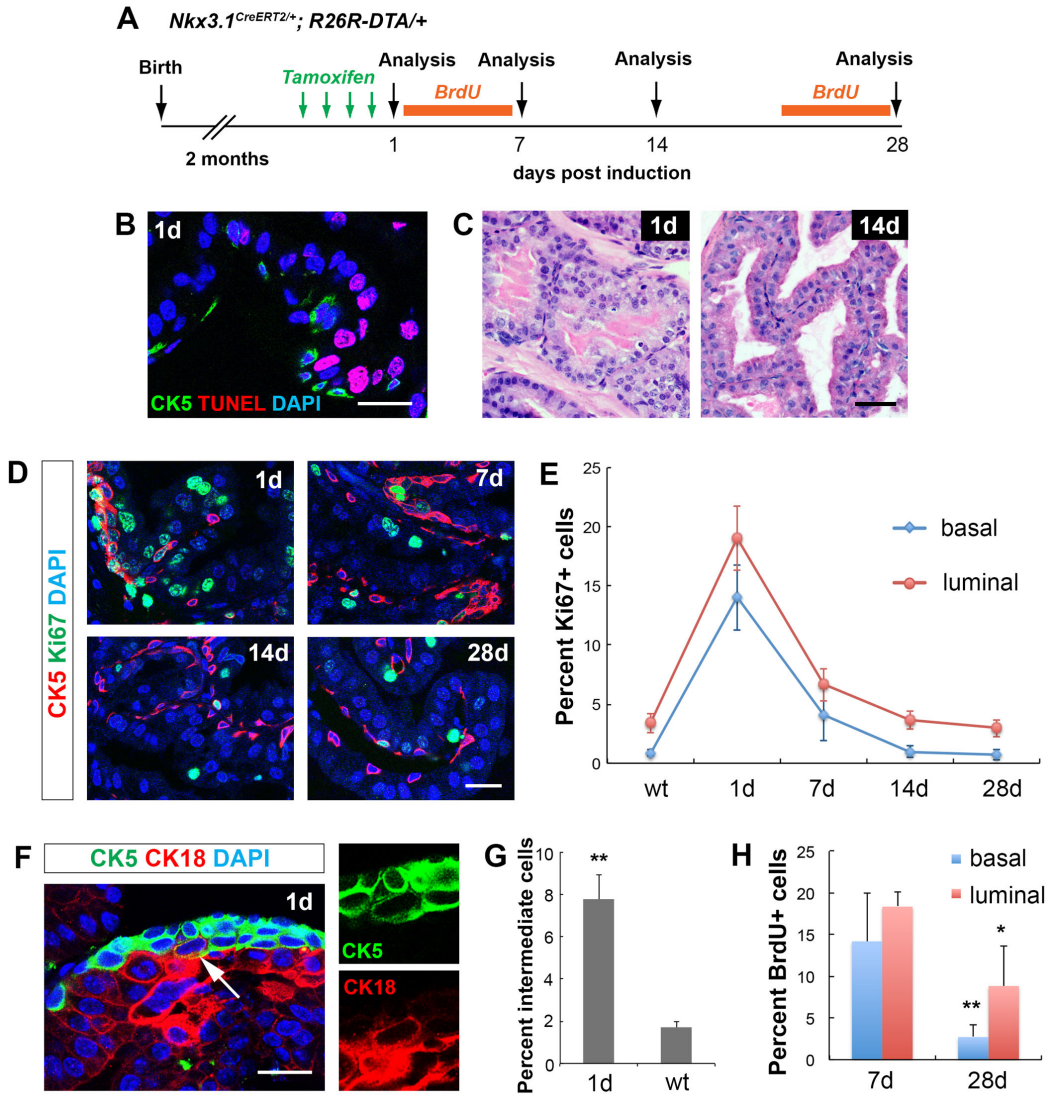
**Cell dynamics during the epithelial repair process upon luminal cell ablation.** (A) Timeline of experiments of luminal cell-specific ablation. (B) IF and TUNEL staining 1 day after tamoxifen induction. Scale bar: 20 μm. (C) H&E images showing 1 day and 14 days prostate epithelium. Scale bar: 50 μm. (D) IF images showing proliferating cells in basal and luminal cells at different time points of tissue repair. Scale bar: 20 μm. (E) Quantitation of Ki67 index in the basal and luminal cell populations at different time points of tissue repair. (F) IF image showing basal cell over-proliferation and CK5^+^CK18^+^ intermediate cells (arrow) at 1 day. Scale bar: 20 μm. (G) Comparison of intermediate cell percentage between 1 day and wild-type control. (H) Quantitation of basal and luminal cell proliferation by BrdU incorporation assay during tissue repair. Error bars in E,G,H correspond to one s.d. *N*=3 animals per time point. **P<0.001; *P<0.01 by two-tailed Student's *t*-test.

After demonstrating an inhibitory role of luminal cells on basal stem cell activities in prostate organogenesis, we next tested whether ablating luminal cells in the adult epithelium reactivates basal cell multipotency. To selectively induce luminal cell death, we used the *Nkx3.1^CreERT2/+^* driver (Wang et al., 2009) to activate the expression of the cytotoxic protein diphtheria toxin A-chain (DTA) from the Rosa26 locus (Wu et al., 2006). *Nkx3.1^CreERT2/+^; R26R^DTA/+^* mice were tamoxifen-induced for 4 consecutive days at 2 months of age, and analyzed 1 day, 7 days, 14 days and 28 days later (Fig. 3A). Mice without tamoxifen induction were used as negative controls. One day after induction, we performed TUNEL and CK5 staining, and found that 34.2% of luminal cells, but not basal cells were apoptotic (Fig. 3B). Disruption of the luminal layer was also visualized by H&E staining (Fig. 3C). Staining with CK5 and Ki67 antibodies revealed significantly increased cell proliferation in both the basal and luminal layers (Fig. 3D,E), which was absent in control mice. Notably, in many foci that presumably had undergone severe luminal cell loss, multiple CK5^+^ cells were stacked into continuous sheath of cells, some of which turned round-shaped and showed a CK5^+^CK18^+^ intermediate cell phenotype (Fig. 3F). The proportion of CK5^+^CK18^+^ intermediate cells among basal cells was 7.8%, significantly higher than the 1.7% in the control (Fig. 3G). We observed similar but more modest phenotypes 7 days post induction. At this stage, stacked basal cells were still present, and were more proliferative than normal, albeit with a reduced rate compared to 1 day (Fig. 3D,E). By 14 days post induction, the prostate displayed normal morphology (Fig. 3C), and the proliferation rates for both basal and luminal cells had decreased back to normal and remained so at 28 days post induction (Fig. 3D,E). The transient burst of basal and luminal cell proliferation upon luminal cell death was also confirmed by performing the BrdU incorporation assays in the first and fourth weeks after tamoxifen induction (Fig. 3A,H). Notably, increased proliferation was not due to inflammation-induced mechanisms (Kwon et al., 2016, 2013), since leukocyte or macrophage levels in the tissue was not elevated 1 day after induction (Fig. S3A,B), and treating the mice with aspirin alongside tamoxifen did not suppress proliferation (Fig. S3C,D). Taken together, our results show that, both basal and luminal cells can promptly respond, via over-proliferation, to the epithelium damage caused by luminal cell death, and that the bulk of the repair process takes place within a week. The increase of CK5^+^CK18^+^ intermediate cells at the apical side of the epithelium support that basal stem cell activities were reactivated to enhance basal-to-luminal differentiation in the repair process, although the possibilities that preexisting intermediate cells underwent expansion or the surviving luminal cells dedifferentiated into an intermediate phenotype cannot be ruled out.

### Dre-rox lineage-tracing of basal cells demonstrates their reactivated multipotency

**Fig. 4.**
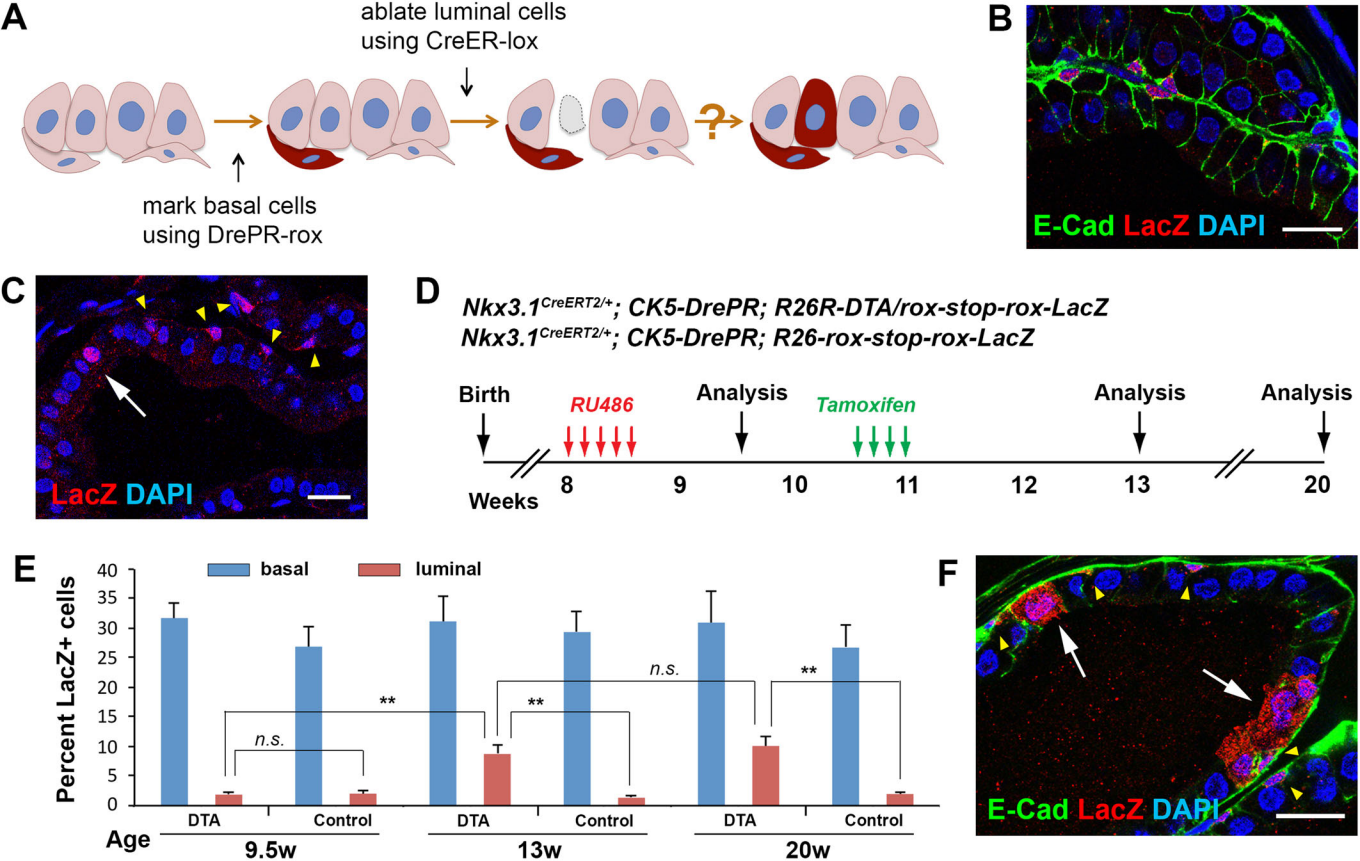
**Lineage-tracing of basal cells upon luminal cell DTA ablation.** (A) Diagram of using the dual-lineage system to trace basal cells. (B) IF showing basal cell labeling upon RU486 treatment of Bas^LacZ^ mice. (C) IF showing a leaked luminal cell (white arrow) that was labeled together with basal cells (yellow arrowheads). (D) Timeline of lineage tracing experiments in Lum^DTA^; Bas^LacZ^ and Lum^WT^; Bas^LacZ^ mice. (E) Quantitation of percentages of LacZ^+^ cells in basal and luminal cells at different time points in Lum^DTA^; Bas^LacZ^ (DTA) and Lum^WT^; Bas^LacZ^ (control) mice. *N*=3 animals per time point in each cohort. Error bar, one s.d. **P<0.001 by two-tailed Student's *t*-test. (F) IF showing LacZ^+^ cell clusters that contained differentiated luminal cells (white arrows). Yellow arrowheads point to LacZ^+^ basal cells in isolation or within the clusters. Scale bars in B,C,F correspond to 20 μm.

To definitively test whether basal-to-luminal cell differentiation occurs upon luminal cell death, we performed basal cell lineage tracing in this context. Since Cre-*loxP* was used for luminal DTA expression, to trace basal cells in the same tissue, we adopted the Dre-*rox* system (Fig. 4A), in which the Dre recombinase can bind to *rox* sites and mediate genomic recombination (Sauer and McDermott, 2004). Dre fused to a progesterone receptor (PR) ligand binding domain was shown to respond to the drug RU486 and could turn on reporter expression in mouse and zebrafish embryos without overlapping activities with Cre (Anastassiadis et al., 2009; Park and Leach, 2013). For marking of adult basal cells, we built a *CK5-DrePR* construct (Fig. S4A), and obtained seven transgenic founder lines upon pronuclear microinjection. The lines were then crossed to the *R26-rox-stop-rox-LacZ* reporter mice (Anastassiadis et al., 2009) to test their efficacy. *CK5-DrePR; R26-rox-stop-rox-LacZ* mice (denoted Bas^LacZ^) were administered with RU486 for 5 consecutive days and analyzed 1 week later. IF staining showed variable degree of basal cell expression and luminal cell leakage for all the lines (Fig. S4B). The best line #21 had robust basal cell DrePR activity with 28.8% of basal cells (*n*=418/1453, three animals analyzed) labeled by LacZ (Fig. 4B), and relatively low luminal cell leakage with 2.1% of luminal cells (*n*=50/2384, three animals analyzed) also labeled (Fig. 4C). This line was chosen for all subsequent experiments.

Next, we generated *Nkx3.1^CreERT2/+^; CK5-DrePR; R26R-DTA/rox-stop-rox-LacZ* mice (denoted Lum^DTA^; Bas^LacZ^) and performed RU486 induction at 8 weeks of age (Fig. 4D). One week later, these mice had the same phenotypes as the Bas^LacZ^ mice and showed no signs of luminal cell apoptosis, confirming the non-overlapping activities of the Cre and Dre systems. The labeling ratios for basal and luminal cells were 31.7% and 1.9%, respectively, consistent with the numbers in the Bas^LacZ^ mice (Fig. 4E). We tamoxifen-induced the Lum^DTA^; Bas^LacZ^ mice 2 weeks after RU486 treatment, and analyzed the prostate 2 weeks and 9 weeks later (Fig. 4D). At 2 weeks post tamoxifen induction (13-weeks-old), we observed that the ratio of LacZ^+^ basal cells remained unchanged (Fig. 4E), indicating LacZ labeling of basal cells was representative. However, the ratio of LacZ^+^ luminal cells over all luminal cells significantly increased to 8.7% (*n*=314/3598, three animals analyzed, *P*=0.0018 by two-tailed Student's *t*-test) (Fig. 4E). No neuroendocrine cells were marked. We concluded that the increase of luminal cell labeling was due to basal-to-luminal differentiation rather than an expansion of the existing leaked luminal cells, because the LacZ^+^ luminal cells were not more proliferative than the unlabeled ones as shown by Ki67 staining (Fig. S4C,D), and because the ratio of labeled luminal cells did not further increase in homeostasis as measured at 9 weeks post tamoxifen induction (20-weeks-old) (Fig. 4E). After epithelial repair, clusters of adjacent cells that comprised both LacZ^+^ basal and luminal cells were present (Fig. 4F), representing clones of reactivated basal stem cells. Importantly, when we performed these experiments using the *Nkx3.1^CreERT2/+^; CK5-DrePR; R26-rox-stop-rox-LacZ* control mice (denoted Lum^WT^; Bas^LacZ^), no increase of LacZ^+^ luminal cell ratios was observed at either 2 weeks or 9 weeks post tamoxifen induction (Fig. 4E), further supporting that ablation of luminal cells triggers the bipotent differentiation of basal cells.

### Basal cell reactivation upon trypsin injection into intact or regressed prostate

**Fig. 5.**
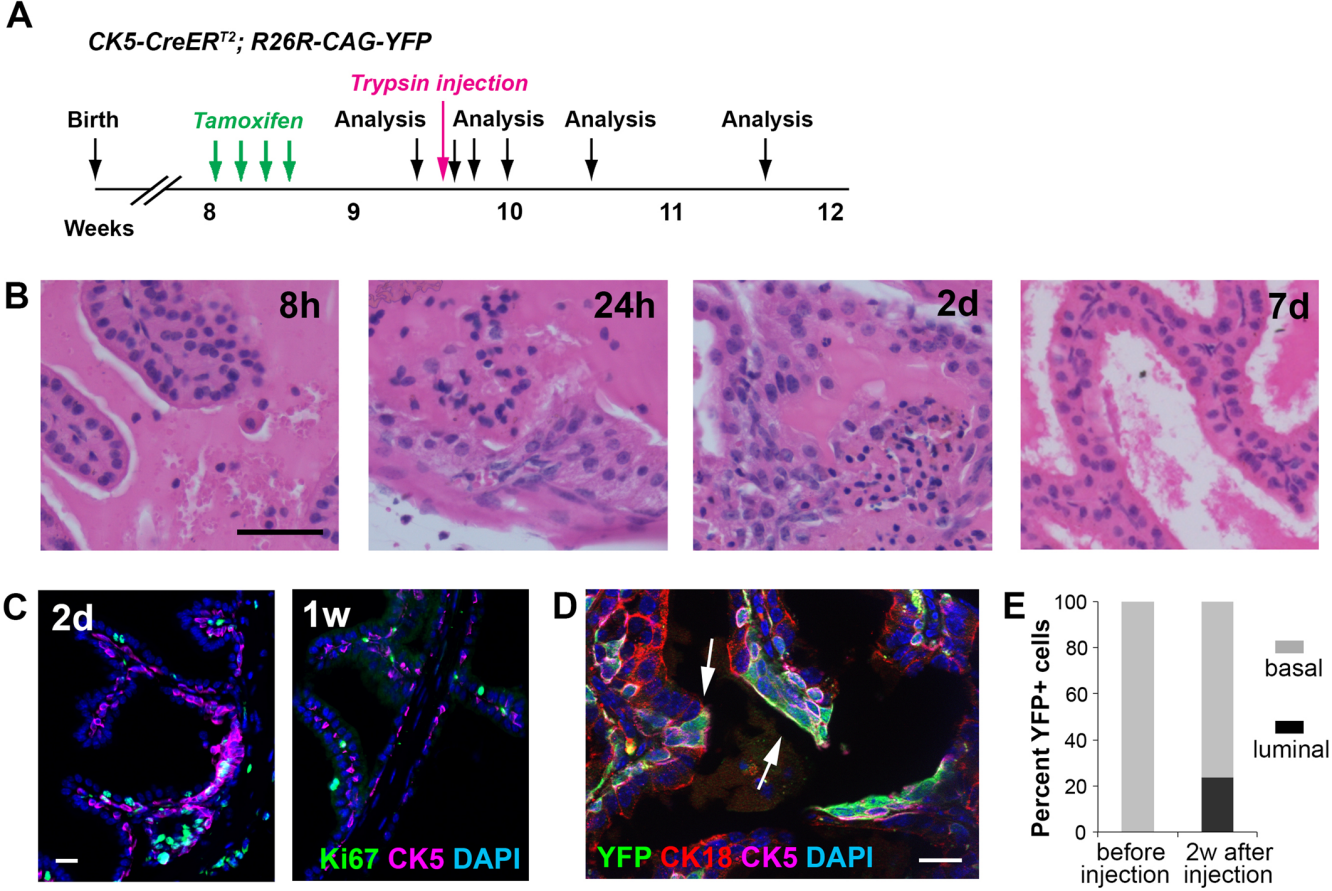
**Lineage-tracing of basal cells upon trypsin-induced luminal cell anoikis.** (A) Timeline of lineage tracing experiment under the trypsin injection condition. (B) H&E images showing luminal cell anoikis and basal cell over-proliferation in the epithelium at early time points after trypsin injection and relatively normal epithelium by 1 week post injection. (C) IF showing proliferating cells 2 days and 1 week after trypsin injection. (D) IF showing YFP^+^ luminal cells (arrows) and repaired epithelium 2 weeks after trypsin injection. (E) Quantitation of basal and luminal proportions of YFP^+^ cells before and 2 weeks after trypsin injection. Scale bars in B correspond to 50 μm, and in C,D to 20 μm.

Besides the DTA-ablation context, we also developed a method to chemically remove luminal cells from the prostate epithelium and test basal cell behaviors using *CK5-CreER^T2^* lineage tracing, which is strictly basal-specific (Wang et al., 2013). Seven days after inducing *CK5-CreER^T2^; R26R-CAG-YFP* mice, we orthotopically injected 30 μl 0.05% trypsin into the prostate (see Materials and Methods), and analyzed prostate morphology 8 h, 1 day, 2 days, 1 week and 2 weeks later (Fig. 5A). We optimized the trypsin dosage so that it could induce significant luminal cell anoikis in localized regions while leaving the basal layer mostly intact (Fig. 5B). Significant luminal cell anoikis was already observed at 8 h post injection and persisted for the first 2 days. Robust cell proliferation was observed at 1 day and 2 days after injection, but was decreased by 1 week, by which time the majority of the epithelium had been repaired (Fig. 5B,C). Importantly, lineage-marked YFP^+^CK18^+^ luminal cells, which were non-existent before trypsin injection, were readily found in clusters 2 weeks after injection (Fig. 5D,E). Therefore, luminal layer damage induced by multiple approaches can stimulate basal-to-luminal cell differentiation for tissue repair.

**Fig. 6.**
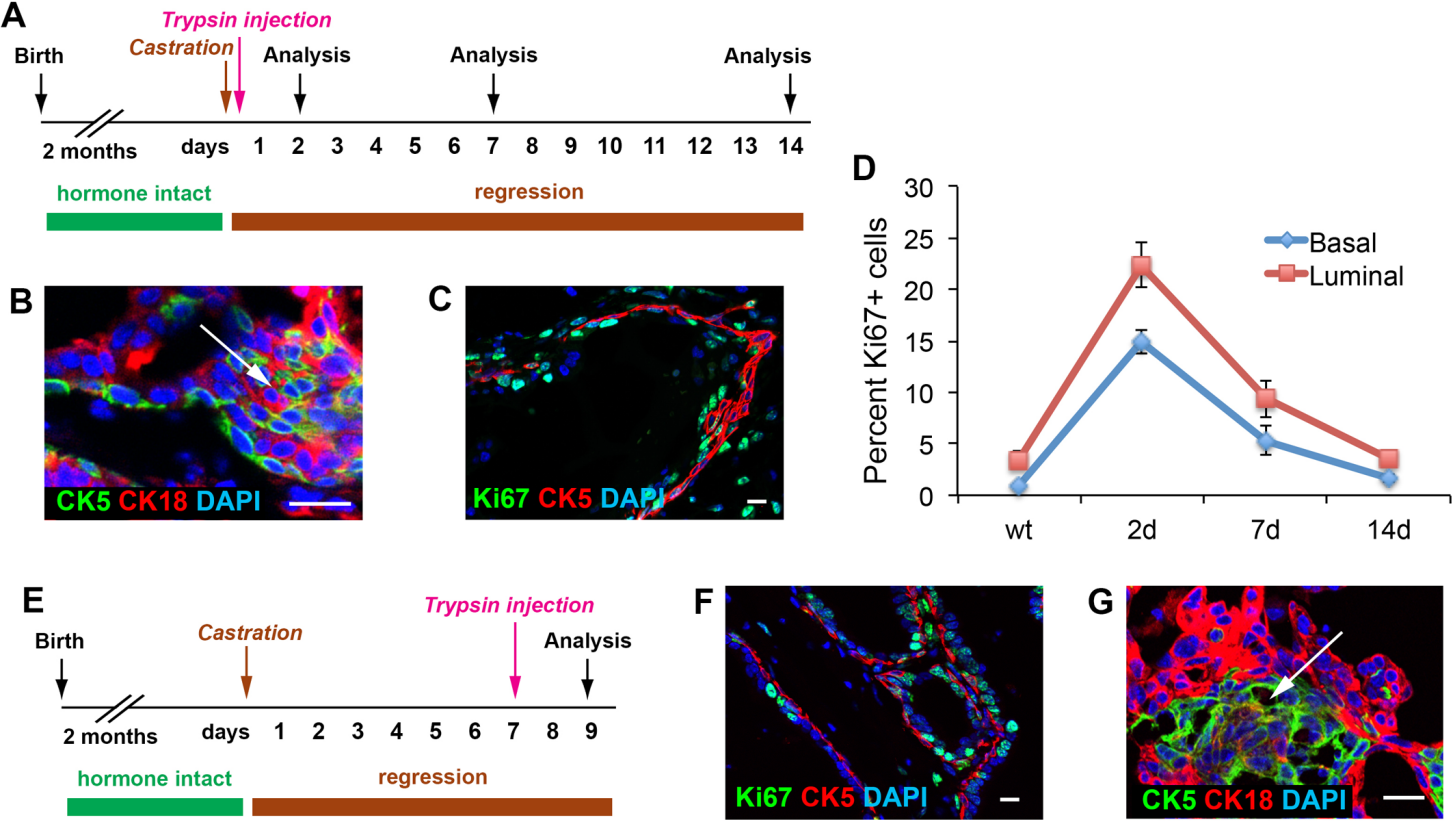
**Analysis of basal cells after trypsin injection in androgen-deprived prostate.** (A) Timeline of experiment of simultaneous trypsin injection and castration. (B,C) IF images showing emergence of intermediate cells (B, arrow) and high proliferation rate in both basal and luminal layers (C) 2 days after trypsin injection. (D) Quantitation of Ki67 index in the basal and luminal cell populations at different time points of tissue repair. *N*=3 animals per time point. Error bars correspond to one s.d. (E) Timeline of experiment of trypsin injection 1 week after mice castration. (F,G) IF images showing high proliferation rate in both basal and luminal layers (F) and emergence of intermediate cells (G, arrow) 2 days after trypsin injection. Scale bars in B,C,F,G correspond to 20 μm.

Finally, we tested whether basal cell reactivation upon luminal layer damage is dependent on androgen, since previous grafting studies showed that androgen could promote basal cell proliferation through stromal paracrine signals (Gao et al., 2001; Hayward et al., 1992). We found that in mice that had undergone castration immediately followed by trypsin injection (Fig. 6A), large numbers of CK5^+^CK18^+^ intermediate cells emerged during the first week of epithelium repair (Fig. 6B). Increased proliferation in basal and luminal cells was also observed (Fig. 6C,D) in similar dynamics to that of hormonally-intact prostates in Fig. 3. In addition, we also performed analysis 2 days after trypsin injection in mice that had undergone prostate regression for 1 week (Fig. 6E), and still observed similar phenotypes (Fig. 6F,G). Therefore, the reactivation of basal cell multipotency upon luminal cell death involves an intrinsic mechanism regulating the prostate epithelial integrity, which is androgen-independent.

## DISCUSSION

Stem cell plasticity is a prominent feature in multiple mammalian organs, since tissue stem cells need to promptly respond to tissue injury while resisting uncontrolled proliferation and differentiation. Both cell-intrinsic transcription factors and external niche signals regulate the decision-making of a tissue stem cell (Blanpain and Fuchs, 2014; Ge and Fuchs, 2018; Wells and Watt, 2018). Unlike some exemplary tissues for studying stem cell plasticity such as skin and small intestine, the mature prostate epithelium is mostly quiescent, and has a relatively simple and rigid architecture of two layers of cells. This may necessitate luminal cells to serve as an important negative niche factor regulating basal stem cell plasticity, counteracting positive stromal signals such as the androgen and Wnt pathways that promote basal cell stemness (Julio et al., 2013; Simons et al., 2012; Xie et al., 2017). This study, using multiple *in vivo* approaches, is the first to definitively show that removal of luminal cells from the adult prostate epithelium induces basal cell multipotency. Our organoid data show that adding luminal cells could directly inhibit basal cell bipotentiality through luminal-basal cell contact. Therefore, the prostate epithelium represents a distinctive paradigm where differentiated daughter cells restrict, rather than support, the multipotency of a tissue stem cell. The mechanisms regulating basal cell reactivation upon luminal cell ablation are likely to be multifaceted. For example, releasing mechanical tension has been shown to promote cell division through Piezo1-dependent pathway (Gudipaty et al., 2017). This might explain the over-proliferation phenotype seen after luminal ablation. It could also explain the different basal cell behaviors between postnatal and adult stages, since young basal cells, particularly those at the budding tip and branching regions, conceivably receive more mechanical stretch and less luminal contact. Alternatively, inhibition might also work through modulation of extracellular matrix and actin cytoskeleton in basal cells to influence their fate decisions, as studies in other tissues have shown that mechanical activation of YAP activity can promote cell stemness (Panciera et al., 2016; Totaro et al., 2017; Yui et al., 2018). Another possibility is that luminal cell contact potentiates TGF-β signaling in basal cells, as TGF-β/SMAD activation has been associated with basal stem cell quiescence in diverse epithelial tissues including prostate (Mou et al., 2016; Valdez et al., 2012). Future research will test these and other possible mechanisms. It will also be interesting to see whether such heterotypic cell–cell interactions occur in other quiescent epithelial tissues as well.

Our findings could have important implications for prostate cancer. It is well established that oncogenic stimuli such as Pten loss in basal cells promote basal stem cell hyper-activation and luminal differentiation (Choi et al., 2012; Wang et al., 2013). How PI3K pathway activation eventually overrides luminal-contact-inhibition awaits further investigation. Harnessing this information may prove to be a promising therapeutic means for intervening with prostate cancer progression, since luminal differentiation from Pten-deleted basal cells is a slow process (Wang et al., 2013), implying a tug of war between the two forces. On the other hand, treatment that kills a fraction of cancer cells might create vacuum for otherwise more dormant cancer cells to be reactivated, if such negative regulation is inherited and preserved in a tumor. Recent example in colon cancer showed that plasticity enabled tumor maintenance despite Lgr5^+^ cancer cell ablation (de Sousa e Melo et al., 2017). It remains to be seen whether analogous mechanism exists in prostate cancer.

## MATERIALS AND METHODS

### Mouse lines and generation of *CK5-DrePR*

The *CK5-CreER^T2^*, *CK18-CreER^T2^*, *Nkx3.1^CreERT2/+^*, *R26R-CAG-YFP*, *Ai9*, *R26R^DTA/+^*, and *R26-rox-stop-rox-LacZ* mouse lines were described previously, or obtained from JAX. The *CK5-DrePR* transgenic line was generated by pronuclear injection of fertilized eggs at Cyagen Biosciences with the *CK5-DrePR* construct, which incorporates a 6.3-kb CK5 promoter (GRCh37/hg19 chr12:52,914,147-52,920,430), a chimeric intron, the DrePR fusion gene sequence (Anastassiadis et al., 2009), and polyA sequence. The *CK5-DrePR* construct was built at GenScript using the pUC57 vector. Animals were maintained in C57BL/6N or mixed background. Genotyping was performed by PCR using tail genomic DNA, with primer sequences listed in Table S1. All animal experiments received approval from the Institutional Animal Care and Use Committee at UCSC.

### Tamoxifen and RU486 induction and aspirin treatment

For tamoxifen induction, mice were administered 9 mg per 40 g body weight tamoxifen (Sigma-Aldrich) suspended in corn oil by oral gavage once daily for 4 consecutive days. For RU486 treatment, mice were administered 1.6 mg per 40 g body weight RU486 (VWR) suspended in corn oil by oral gavage once daily for 5 consecutive days. For aspirin treatment, O-Acetylsalicylic acid (Thermo Fisher Scientific, Cat No. AC158180500) was dissolved in equimolar NaHCO_3_ solution, and administered through oral gavage at 20 mg once daily. Treatment started 1 day before tamoxifen induction, and lasted 5 consecutive days.

### Prostate orthotopic trypsin injection

For luminal cell detachment from the prostate epithelium, trypsin solution was orthotopically injected into anesthetized mice. Briefly, a horizontal incision was made at the abdomen above the bladder and the seminal vesicles with their attached anterior prostate lobes were exposed. 30 μl 0.05% trypsin was then slowly injected into the prostate lobes using syringe (Hamilton 7656-01) and 33-gauge 0.5 inch Hamilton needle (Cat No. 89221-012) with the assistance of anatomical lens, before the incision was sutured.

### BrdU incorporation assay

BrdU (Sigma-Aldrich) was dissolved in PBS (10 mg/ml) and administered by intraperitoneal injection twice daily (0.1 ml per dose) for 7 consecutive days during tissue repair to label proliferating cells.

### Prostate dissociation and flow cytometry

To isolate prostate basal and luminal cells, prostate tissues were dissected and minced to small clumps, followed by enzymatic dissociation with 0.2% collagenase I (Invitrogen) in DMEM media with 5% FBS for 3 h at 37°C. Tissues were digested with 0.25% Trypsin-EDTA (StemCell Technologies) for 1 h at 4°C, passed through 21- to 26-gauge syringes and filtered through a 40-μm cell strainer to obtain single-cell suspensions. Dissociated prostate cells were suspended in Hanks' Balanced Salt Solution Modified/2% FBS. ROCK inhibitor Y-27632 (StemCell Technologies) was added at 10 μM throughout the whole process to inhibit luminal cell death. Lineage-marked basal and luminal cells were sorted based on YFP and tdTomato positivity, respectively. Antibodies used for sorting of Trop2^+^ basal cells and Lin^−^Sca-1^+^CD49f^hi^ basal cells, leukocytes, and macrophages are listed in Table S2. Sorting was performed on a BD FACS Aria II instrument in the Flow Cytometry Shared Facility of UCSC.

### Tissue reconstitution/renal graft assay

Flow-sorted basal and luminal cells were mixed at different ratios and then mixed with 2.5×10^5^ dissociated urogenital sinus mesenchyme (UGM) cells from embryonic day 18.0 rat embryos. UGM cells were obtained from dissected urogenital sinus treated for 30 min in 1% trypsin, followed by mechanical dissociation and treatment with 0.1% collagenase B (Roche) for 30 min at 37°C, and washing in PBS. Pelleted cell mixtures were resuspended in 10 μl of 9:1 collagen/setting buffer [10× Earle's Balanced Salt Solution (Life Technologies), 0.2 M NaHCO_3_ and 50 mM NaOH], and gelatinized at 37°C for 20 min. Tissue recombinants were cultured in DMEM/10% FBS supplemented with 10^−7^ M DHT overnight, followed by transplantation under the kidney capsules of immunodeficient NCRNU-M sp/sp nude mice (Taconic Biosciences). Grafts were collected after 2 months of growth and imaged under a Nikon SMZ-1000 stereomicroscope with fluorescence and charge-coupled device digital camera. YFP^+^ graft areas were quantified using ImageJ.

### Prostate organoid culture

Flow-sorted basal and luminal cells were washed with advanced DMEM/F12 (Life Technologies), and resuspended in 10 μl advanced DMEM/F12 and 30 μl Matrigel per well in the Nunc Lab-Tek II CC2 Chamber Slide System (Thermo Fisher Scientific). Chamber slide was put upside down in the 37°C cell culture incubator for 15 min to let the matrigel solidify. Mouse prostate organoid culture medium was prepared according a previous protocol (Drost et al., 2016). Briefly, the following components were added to advanced DMEM/F12 medium, B27 (50× diluted), HEPES 1 M (100× diluted), GlutaMAX (100× diluted), Penicillin-streptomycin (100× diluted), N-acetylcysteine (1.25 mM), EGF (50 ng/ml), A83-01 (200 nM), Noggin (100 ng/ml), R-spondin 1 (500 ng/ml), DHT (1 nM), Y-27632 dihydrochloride (10 μM). Organoid culture medium was pre-warmed before adding to the wells. The medium was changed with fresh standard medium or paralleled luminal condition medium every 3 days. Organoids were fixed in 4% PFA for 20 min at room temperature before immunofluorescence staining. *In situ* organoid images were taken using the Keyence microscope in the UCSC Microscopy Shared Facility. Organoid sizes were quantified using ImageJ.

### Histology and immunofluorescence staining

H&E staining was performed using standard protocols as previously described (Xie et al., 2017), and visualized using a Zeiss Axio Imager. Immunofluorescence staining was performed using 6 μm cryosections or on organoids *in situ*. Samples were incubated with 10% normal goat serum (NGS) and primary antibodies diluted in 10% NGS overnight at 4°C. Samples were then incubated with secondary antibodies (diluted 1:500 in PBST) labeled with Alexa Fluor 488, 555, or 647 (Invitrogen/Molecular Probes). Slides were mounted with VectaShield mounting medium with DAPI (Vector Labs), and images were taken on a Leica TCS SP5 spectral confocal microscope in the UCSC Microscopy Shared Facility. All primary antibodies and dilutions used are listed in Table S2.

### TUNEL assay

TUNEL assay was performed using the In Situ Cell Death Detection Kit, TMR Red or Fluorescein (Sigma-Aldrich) according to the manufacturer’s instructions with modifications. Briefly, slides were incubated in freshly prepared permeabilization solution (0.1% Triton X-100 in 0.1% sodium citrate) for 2 min on ice, washed with PBS twice, and then incubated with TUNEL reaction mixture (1:20 terminal deoxynucleotidyl transferase solution diluted in label solution) for 40 min at 37°C in a dark humidified chamber. Immunofluorescence staining with CK5 or CK18 was performed immediately afterwards as described above. Signals were detected using the Leica TCS SP5 spectral confocal microscope.

### Lineage analysis and statistics

For lineage-tracing analysis, cell numbers were counted manually using confocal ×40 photomicrographs across tissue sections. Basal cells were identified based on the oval or triangular shape of the cells, their positions at the basement of the epithelium, and positive CK5 staining. Luminal cells were determined based on the columnar shape of the cells, their positions at the apical side of the epithelium, and positive CK18 staining. Statistical analyses were performed using the Mann–Whitney *U*-test, or two-tailed Student's *t*-test as appropriate. At least three animals for each experiment or genotype were analyzed. No statistical methods were used to pre-determine sample size, and animals were not randomized. Investigators were blinded to the animal IDs when analyzing phenotypes. The variances were similar between the groups that were being statistically compared.

## Supplementary Material

Supplementary information
