# Supplementary material for: Luminal-contact-inhibition of epithelial basal stem cell multipotency in prostate organogenesis and homeostasis
Source: Biol Open. 2019 Sep 20;8(10):bio045724. doi: 10.1242/bio.045724 (PMC6826291; doi:10.1242/bio.045724)
Supplement: Supplementary information [file biolopen-8-045724-s1.pdf]

Supplemental Figures and Tables

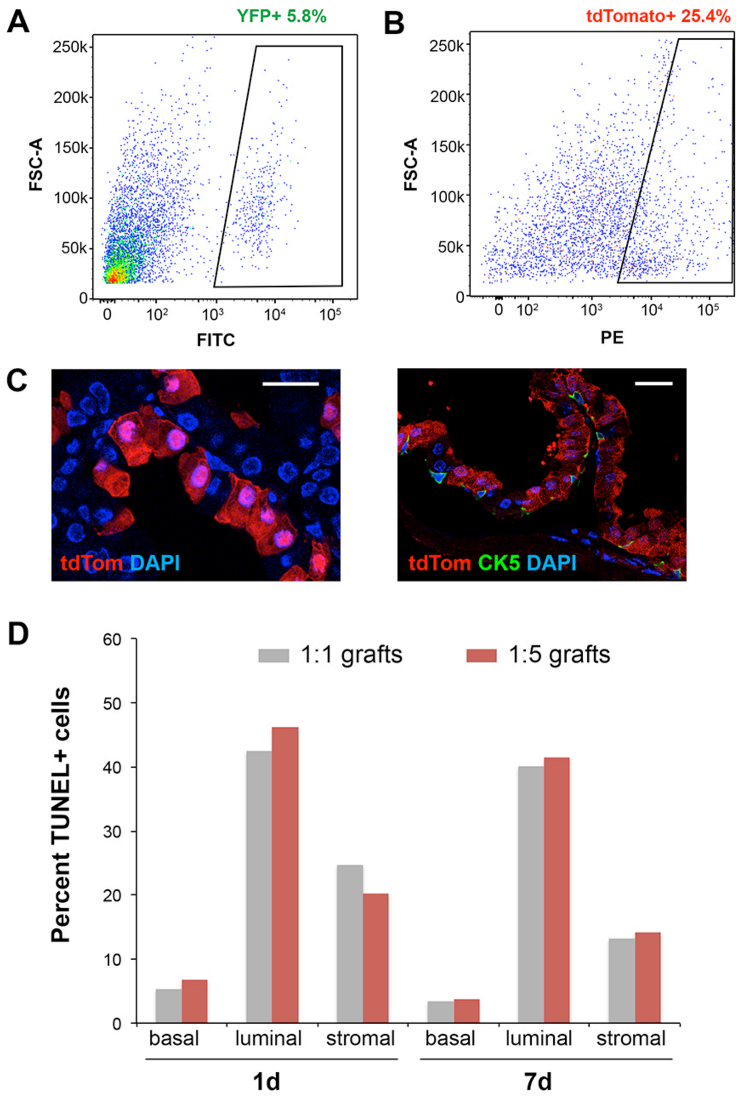

Figure S1. Sorting basal and luminal cells for renal grafting. (**A**, **B**) FACS gating for YFP^+ basal cells (A) and $tdTomato^+$ luminal cells (B) from induced *CK5-CreER*^{T2}*; R26R-CAG-YFP/+* mice and *CK18-CreER*^{T2}*; Ai9/+* mice, respectively. (**C**) Direct visualization of tdTomato and IF staining with CK5 antibody in *CK18-CreER*^{T2}*; Ai9/+* mice showing specific marking of luminal cells after induction. Scale bars, 20 µm. (**D**) Quantitation of apoptotic cells per cell type for all the 1:1 grafts and 1:5 grafts at 1d and 7d post renal grafting.

Biology Open • Supplementary information

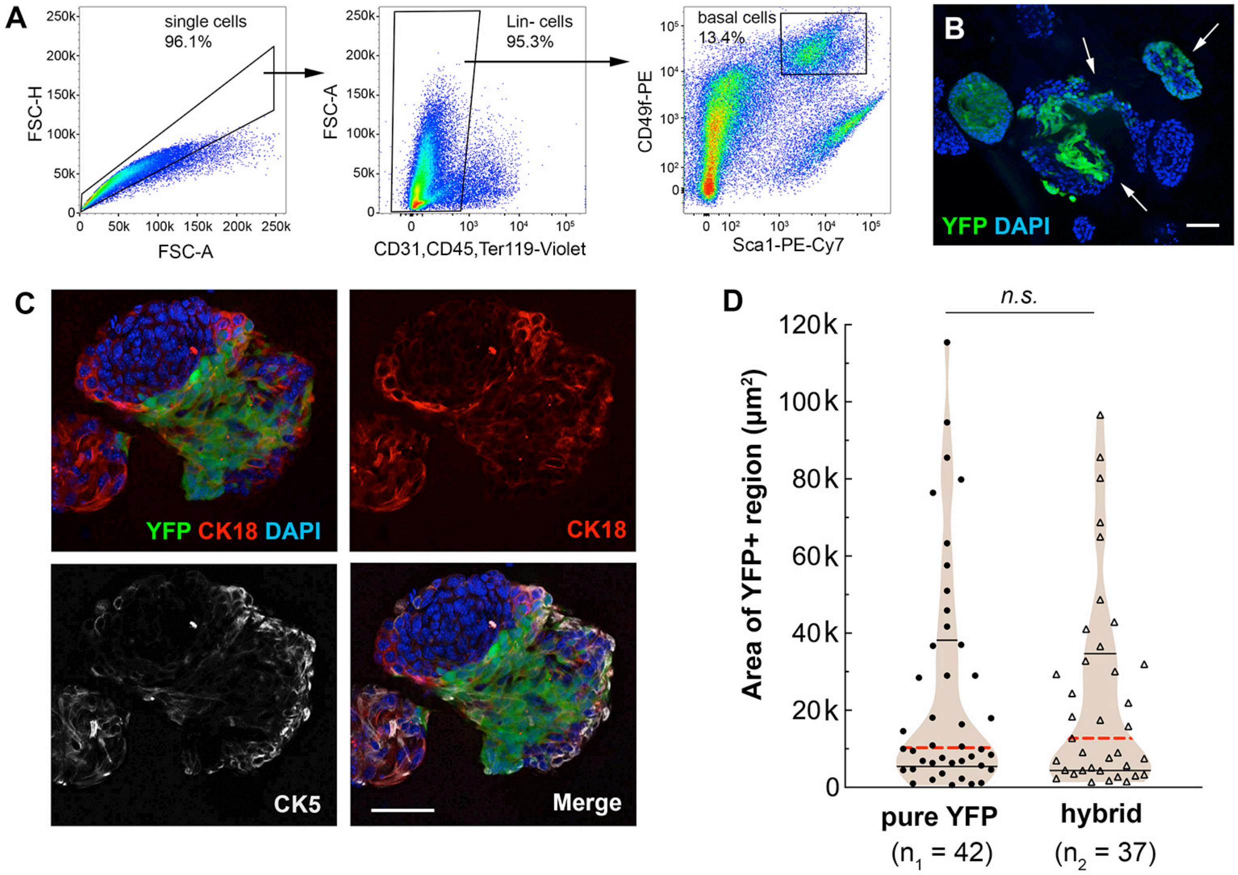

Figure S2. Organoid assay with mixture of YFP+ basal cells and unmarked basal cells. (**A**) FACS gating for sorting average basal cells based on $Lin^{-}Sca\text{-}1^{+}Cd49f^{hi}$. (**B**) Direct visualization of YFP in organoids derived from green and white basal cell mixture. Arrows point to green-white basal hybrid organoids. Scale bar, 50 µm. (**C**) IF staining showing YFP+ basal cells can differentiate into luminal cells in the hybrid organoids. Scale bar, 50 µm. (**D**) Violin plot comparing area of YFP+ regions between pure-YFP^{+}-basal-derived organoids and hybrid organoids. Black lines, quartiles. Red dashed lines, medians. n.s., not significant by Mann-Whitney U Test.

Biology Open • Supplementary information

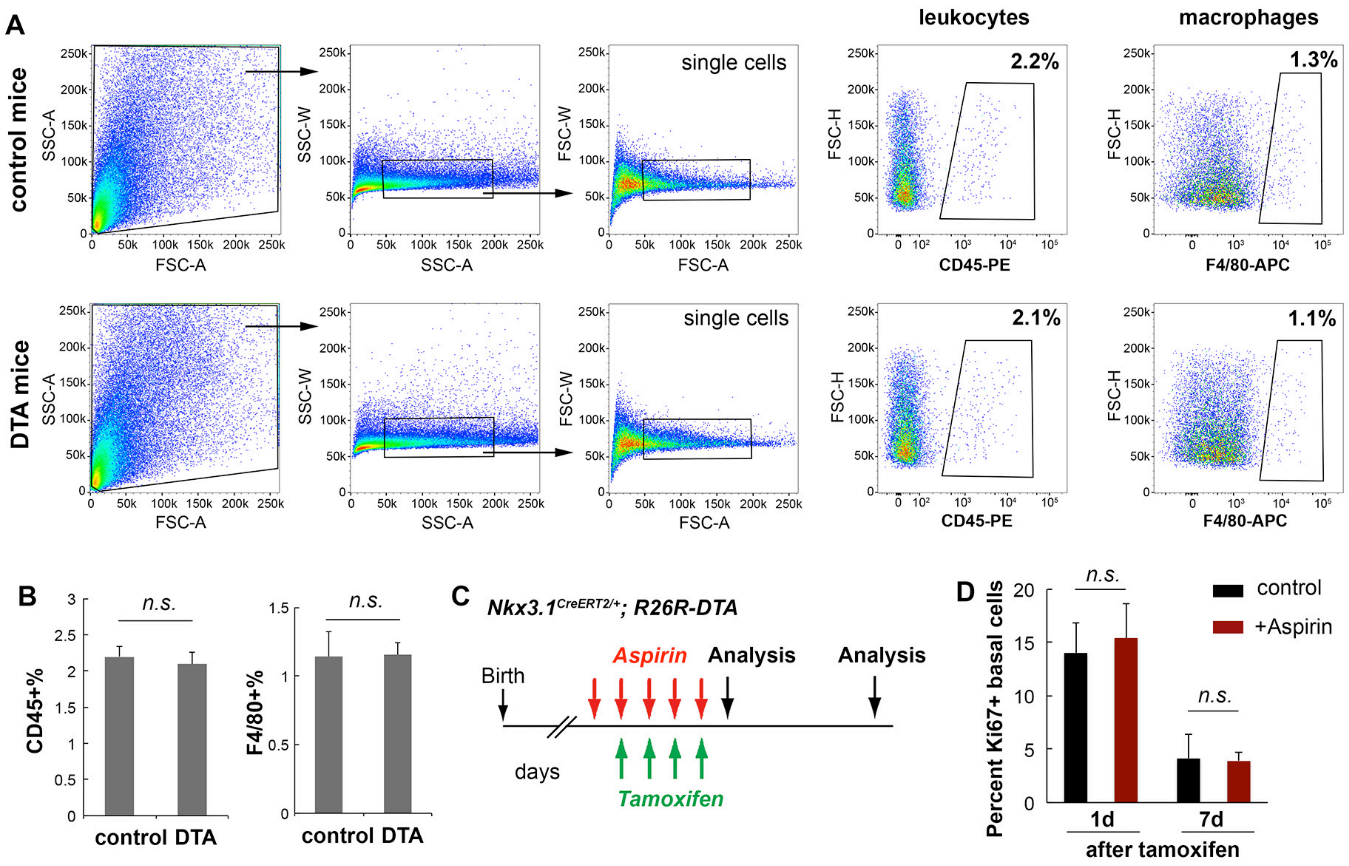

Figure S3. Testing potential influence of inflammation response on basal cell proliferation. (**A**) FACS gating for sorting CD45$^+$ leukocytes and F4/80$^+$ macrophages from control un-induced mice and *Nkx3.1*$^{CreERT2/+}$*; R26R*$^{DTA/+}$ mice one day after induction. (**B**) Quantitation of percentage of leukocytes and macrophages among prostate cells in control and induced *Nkx3.1*$^{CreERT2/+}$*; R26R*$^{DTA/+}$ mice. N=3 biological replicates. n.s., not significant by student's t-test. (**C**) Timeline of aspirin treatment experiment. (**D**) Quantitation of percentage of proliferating basal cells showing aspirin treatment had no effect both 1d and 7d after induction. N=3 biological replicates per group. n.s., not significant by student's t-test.

Biology Open • Supplementary information

A

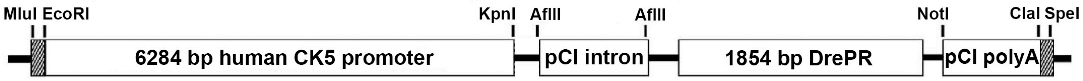

B

CK5-DrePR line	basal cell expression	luminal cell expression
#16	+++	++
#21	+++	+
#24	+	++
#27	+	++
#34	++	++
#42	++	+
#43	+++	++

C

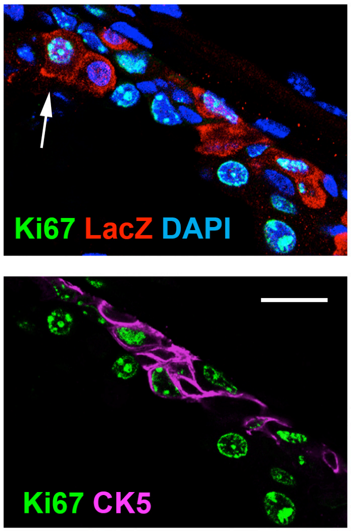

D

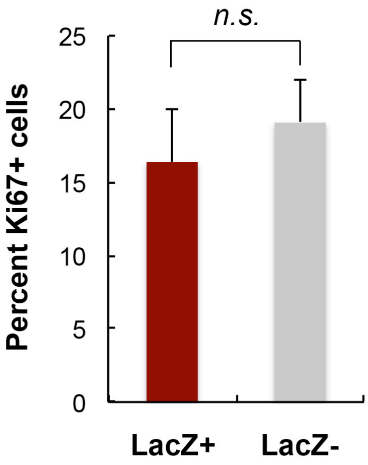

Figure S4. Generation and characterization of the Dre-rox lineage tracing system. (**A**) Schematic diagram of the *CK5-DrePR* construct for generation of transgenic mice. (**B**) Table summary of expression levels in prostate basal and luminal cells for all the *CK5-DrePR* founder lines. "+++", strong and high-frequency expression; "+", sporadic expression. (**C**) Representative IF image showing presence of Ki67^{+} cells in both LacZ+ (arrow) and LacZ^{-} luminal cells 1d after tamoxifen induction in Lum^{DTA}; Bas^{LacZ} mice. Scale bar, 20 μm. (**D**) Quantitation of Ki67 index between the LacZ^{+} and LacZ^{-} luminal cells. N=3 animals per group. n.s., not significant by student's t-test.

Biology Open • Supplementary information

Table S1. Primers for mouse genotyping.

**Allele**		**Primer sequence**
CreER^{T2}	forward	5'-CAGATGGCGCGGCAACACC-3'
	reverse	5'-GCGCGGTCTGGCAGTAAAAAC-3'
	reverse	5'-GTCATCTTCACTTAGCCATTGG-3'
R26R-CAG-YFP	wild-type forward	5'-AAGGGAGCTGCAGTGGAGTA-3'
	wild-type reverse	5'-CCGAAAATCTGTGGGAAGTC-3'
	mutated forward	5'-ACATGGTCCTGCTGGAGTTC-3'
	mutated reverse	5'-GGCATTAAGCAGCGTATCC-3'
Nkx3.1	wild-type forward	5'-CTCCGCTACCCTAAGCATCC-3'
	wild-type reverse	5'-GACACTGTCATATTACTTGGACC-3'
Ai9	mutated forward	5'-CTGTTCCTGTACGGCATGG-3'
	mutated reverse	5'-GGCATTAAAGCAGCGTATCC-3'
R26R-DTA	forward	5'-AAAGTCGCTCTGAGTTGTTAT-3'
	wild-type reverse	5'-GGAGCGGGAGAAATGGATATG-3'
	mutated reverse	5'-GCGAAGAGTTTGTCCTCAACC-3'
R26-rox-stop-rox-LacZ	forward	5'-TGGAAATGTTACCAAGGAACT-3'
	wild-type reverse	5'-GCTTTAAAGAAAGCCCACAG-3'
	mutated reverse	5'-TGACAGGAGATCCTGCCCCGGCACT-3'
DrePR	forward	5'-CATTCACAGCTCGACAGCTC-3'
	reverse	5'-TGAACTGGGAGTGGACACCT-3'

Biology Open • Supplementary information

Table S2. Antibodies used in this study

Antibodies for flow cytometry

Antibody	Supplier	Dilution
Sca-1-PE-Cy7	Biolegend clone E13-161.7 #122513	1:500
CD49f-PE	eBiosciences clone eBioGoH3 #12-0495	1:300
Ter119-eFluor450	eBiosciences clone Ter-119 #48-5921	1:250
CD31-eFluor450	eBiosciences clone 390 #48-0311	1:250
CD45-eFluor450	eBiosciences clone 30-F11 #48-0451	1:250
CD45-PE	eBiosciences clone 30-F11 #12-0451-82	1:250
Trop2- Biotinylated	R&D Systems #BAF1122	1:140
APC Streptavidin	BD #554067	1:140
F4/80-APC	Biolegend #123115	1:250

Primary antibodies used for immunofluorescence staining

Antigen	Supplier	Ig type	Dilution
BrdU	Serotec #MCA2060	rat IgG2a	1:500
CK5	Covance #PRB-160P	rabbit IgG	1:1000
CK18	Abcam #ab668	mouse IgG1	1:100
E-Cadherin	BD Biosciences #610181	mouse IgG2a	1:500
Ki67	DakoCytomation #M7249	rat IgG2a	1:600
LacZ	Fisher #PA1-21477	rabbit IgG	1:2000
Synaptophysin	Zymed #18-0130	rabbit IgG	1:500
YFP	Abcam #13970	chick IgY	1:2000

Biology Open • Supplementary information
